# Role of pyroptosis in hemostasis activation in sepsis

**DOI:** 10.3389/fimmu.2023.1114917

**Published:** 2023-01-23

**Authors:** Chengrui Zhu, Yingjian Liang, Yangtuo Luo, Xiaochun Ma

**Affiliations:** ^1^ Department of Critical Care Medicine, The First Hospital of China Medical University, Shenyang, Liaoning, China; ^2^ Department of Otolaryngology, The First Hospital of China Medical University, Shenyang, Liaoning, China

**Keywords:** pyroptosis, inflammasome, coagulation, hemostasis, sepsis, tissue factor

## Abstract

Sepsis is frequently associated with hemostasis activation and thrombus formation, and systematic hemostatic changes are associated with a higher risk of mortality. The key events underlying hemostasis activation during sepsis are the strong activation of innate immune pathways and the excessive inflammatory response triggered by invading pathogens. Pyroptosis is a proinflammatory form of programmed cell death, that defends against pathogens during sepsis. However, excessive pyroptosis can lead to a dysregulation of host immune responses and organ dysfunction. Recently, pyroptosis has been demonstrated to play a prominent role in hemostasis activation in sepsis. Several studies have demonstrated that pyroptosis participates in the release and coagulation activity of tissue factors. In addition, pyroptosis activates leukocytes, endothelial cells, platelets, which cooperate with the coagulation cascade, leading to hemostasis activation in sepsis. This review article attempts to interpret the molecular and cellular mechanisms of the hemostatic imbalance induced by pyroptosis during sepsis and discusses potential therapeutic strategies.

## 1 Introduction

Sepsis is defined as a life-threatening condition caused by a dysregulated host response to infection (1). It has been recognized as a global health priority by the World Health Organization and is the most common cause of death in hospitals, causing 11 million deaths worldwide per year (2). During sepsis, the invading pathogen encounters the host innate immune system triggering a sustained systemic inflammatory response and coagulation cascade. The co‐dependency of innate immune pathways and the hemostasis process is termed “immunothrombosis” or “immunocoagulation” (3). The “immunothrombosis” process serves as the non-specific first line of host defense against pathogens. However, the over-activation of the hemostatic system leads to excessive thrombin formation and hyperfibrinolysis, resulting in disseminated intravascular coagulation (DIC) and ultimately to multiple organ dysfunction and septic death (4).

Pyroptosis, a recently discovered programmed mode of cell death, participates in the innate immune response, inhibiting the replication of intracellular pathogens, and activating immune cells to phagocytose and kill pathogens (5, 6). However, overactivated pyroptosis leads to dysregulated host immune response and organ dysfunction (7, 8). Pyroptosis occurs in many cell types and participates in the pathophysiological processes of sepsis (9). More recently, pyroptosis has been shown to play a prominent role in the prothrombotic response in sepsis (10, 11). This review article attempts to interpret the molecular and cellular mechanisms of the hemostatic imbalance induced by pyroptosis in sepsis and discusses potential therapeutic strategies.

## 2 Process of hemostasis and thrombosis

**Figure 1:**
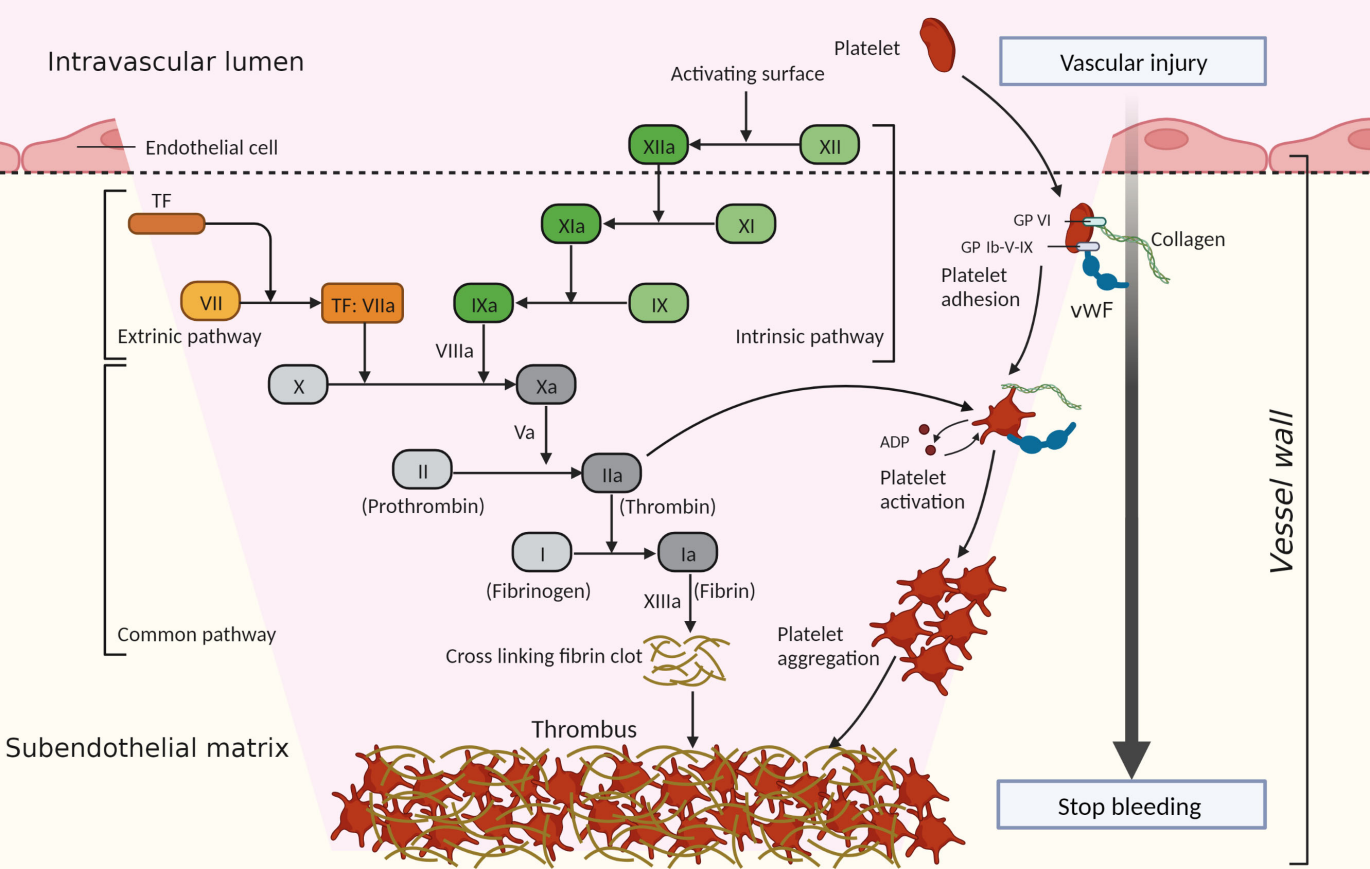
Process of hemostasis and thrombosis. Hemostasis and thrombosis share the core pathways of platelet activation and the coagulation cascade, which act together to generate a hemostatic clot or thrombus. Platelets are recruited to a site of vessel injury where collagen is exposed through the interaction of cell-surface receptors (such as glycoprotein VI [GP VI], glycoprotein Ib-V-IX [GP Ib-V-IX], and some integrins [not shown]) with the collagen and released von Willebrand factor (vWF). Then platelets are activated and adhere to the vessel wall, releasing agonists (such as adenosine diphosphate [ADP]), which promote continued platelet activation and aggregation. The extrinsic pathway is initiated by exposed tissue factor (TF, also known as factor III). TF forms a complex with circulating factor VIIa, thereby triggering the coagulation cascade. The intrinsic pathway begins with the contact of factor XII with the negatively charged surface. Factor XII is activated and triggers a chain reaction of coagulation factors. When coagulation factor X is activated through the intrinsic or extrinsic pathways, it converts prothrombin (factor II) to thrombin (factor IIa). Thrombin further cleaves fibrinogen (factor I) into fibrin (factor Ia), forming a mesh that binds to and strengthens the platelet clot and stops bleeding.

Hemostasis is an important physiological process that prevents bleeding after a blood vessel injury and involves two main mechanisms: blood coagulation and platelet activation. Thrombosis is generally considered to be the pathological progression of hemostasis, and refers to thrombus formation inside blood vessels, resulting in the occlusion of arteries, veins, and microvessels (12). It is a critical event in many diseases, including myocardial infarction, stroke, venous thromboembolism, and DIC. Hemostasis and thrombosis share the core pathways of platelet activation and the coagulation cascade, and the processes will be briefly described to better understand the underlying mechanisms of hemostasis imbalance in sepsis (**Figure 1**).

When blood vessels are disrupted, collagen in the subendothelial matrix and tissue factor (TF) are exposed to the blood, which initiates thrombus formation (12–14). Platelets are recruited to the injury sites through the efficient interaction of cell-surface receptors with the exposed collagen and released von Willebrand factor (vWF) in the subendothelial matrix (15), then the platelets become activated. In addition, exposed TF also initiates platelet activation by generating thrombin through the coagulation cascade (16). Thrombin cleaves protease-activated receptor 4 in platelets and activates the platelets (17). After the recruitment and activation stages, platelets firmly adhere to the vessel wall and promote continued platelet activation and aggregation, resulting in rapid thrombus growth (12).

In parallel with platelet recruitment, the coagulation cascade is initiated to stabilize and reinforce the platelet thrombus (12). The coagulation cascade, first described in 1964, involves the sequential activation of a series of plasma serine proteases in the generation of activated thrombin (18, 19). The classical view of the “cascade” model distinguishes two initiation pathways: the extrinsic pathway and the intrinsic pathway. The extrinsic pathway is initiated by TF (also known as factor III), which is expressed by vessel-wall cells that are not exposed to blood and by tissue cells (20). With TF exposure to blood, it binds to factor VIIa and triggers the coagulation cascade (13). The TF-initiated pathway is activated by trauma, infection, or the disruption of the vessel wall under normal hemostasis (13, 21). The intrinsic pathway of coagulation, also known as the contact pathway, begins upon exposure of the “contact” factor (factor XII) in plasma to a negatively charged surface (18). The exposed endothelial cells, platelets, collagen, and foreign surfaces, as acting surfaces, initiate factor XII activation and trigger a chain reaction of coagulation factors (18). However, this pathway is not linked to normal hemostasis, and instead contributes predominantly to pathological thrombosis (22). When coagulation factor X is activated through the intrinsic or extrinsic pathways, it converts prothrombin to thrombin (19). Thrombin further cleaves fibrinogen into fibrin, forming a mesh that binds to and strengthens the platelet clot and stops bleeding. This is known as the common pathway (18, 19, 22).

During hemostasis and thrombosis, platelet thrombus formation and fibrin deposition occur concomitantly and are connected with each other (3). Under normal conditions, regulatory mechanisms of anticoagulation (such as tissue factor pathway inhibitor [TFPI], protein C system, antithrombin [AT]) and fibrinolysis systems restrict blood clot formation in a temporary and localized manner (23). However, when pathological processes overwhelm the regulatory mechanisms of hemostasis, thrombosis is initiated (12), occurring as arterial and venous thrombosis or DIC.

## 3 Hemostasis activation in sepsis

During sepsis, pathogens trigger an impaired host response, leading to innate immune response, systemic inflammation, and eventually activation of the coagulation response (24, 25). Clinically, almost all patients with sepsis have hemostasis abnormalities, ranging from slight coagulation marker changes to stronger coagulation activation with a decrease in platelet counts and prolongation of coagulation time, to fulminant coagulation activation with widespread micro-thrombosis and profuse bleeding, known as DIC (26).

**Figure 2:**
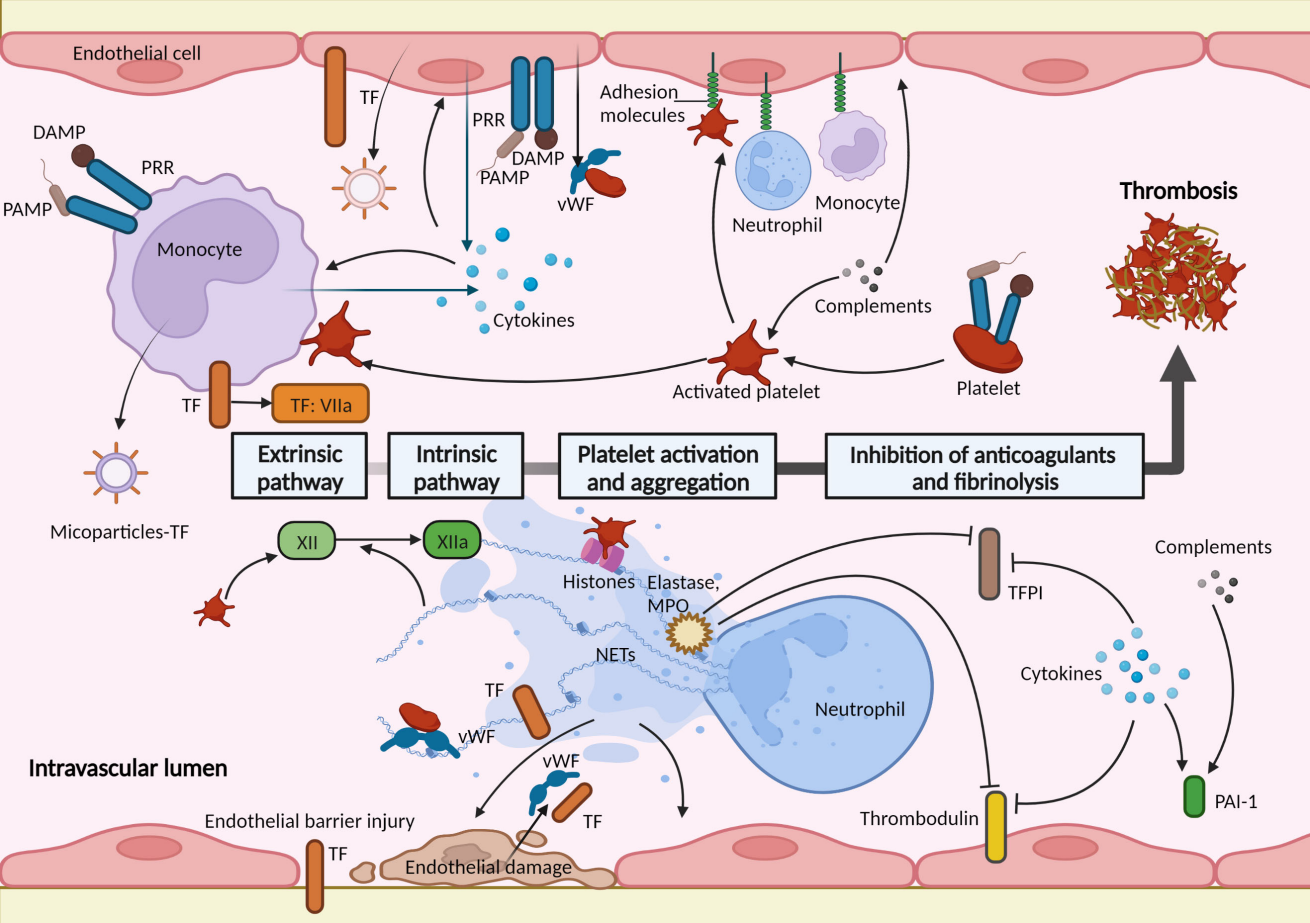
Immune-related factors in hemostasis activation during sepsis. Activation of the coagulation pathway, platelet activation and aggregation, and impaired anticoagulant and fibrinolytic pathways act cumulatively to a procoagulant state in sepsis. These are mediated by multiple immune-related molecular and cellular factors. Monocytes and endothelial cells play important roles in thrombus formation during sepsis. They express and release tissue factor (TF) when recognizing pathogen-associated molecular patterns (PAMPs) or damage-associated molecular patterns (DAMPs) through pattern recognition receptors (PRRs). The activated endothelial cells serve as a bridge between the coagulation response and immune factors. Endothelial cells expressed adhesion molecules (such as E-selectin and intercellular adhesion molecule 1) mediating the adhesion and interactions of immune cells. The injured endothelial barrier loses antithrombotic function and induces exposure of TF; the damaged endothelial cells release prothrombotic molecules, such as von Willebrand factor (vWF) and TF. Platelets are activated and interact with circulating leukocytes and endothelial cells, and trigger factor XII initiating the intrinsic coagulation pathway. Neutrophil extracellular traps (NETs), released by neutrophils, support immunothrombosis in several ways: activate and damage endothelial cells; NETs and histones in NETs promote the recruitment and activation of platelets; bind to TF and activate factor XII; neutrophil elastase and myeloperoxidase (MPO) in NETs inhibit the anticoagulants. Activated complements activate endothelial cells and platelets and increase the level of plasminogen activator inhibitor-1 (PAI-1). Proinflammatory cytokines increase the expression of TF in monocytes and endothelial cells; activate leukocytes, platelets, and endothelial cells; downregulate anticoagulants and fibrinolysis pathways. TFPI, tissue factor pathway inhibitor.

In general, blood cells, that is, monocytes, neutrophils, platelets, and vascular endothelial cells, together with coagulation factors, play significant roles in hemostatic changes in sepsis (24, 27). Moreover, in addition to increased coagulation, the hemostatic change also involves the downregulation of anticoagulant mechanisms and an impaired fibrinolytic response (24). Importantly, the key events underlying the hemostasis activation in sepsis are the strong activation of innate immune cells and an excessive inflammatory response (23). “Immunothrombosis” has been therefore introduced to emphasize the pivotal role of the innate immune response in hemostasis activation and thrombosis, which can protect against pathogens in the vascular system and are also involved in pathological thrombosis (3). Multiple immune-related molecular and cellular factors contribute to hemostasis activation in sepsis, which is generally supported by distinct cells and molecules that are irrelevant to physiological hemostasis. The roles of innate immune cells and immune-related factors in hemostasis activation in sepsis are summarized below (**Figure 2**).

### 3.1 Tissue factor

The most principal initiator of coagulation activation in sepsis is TF (24). TF is a transmembrane protein constitutively expressed by vessel wall cells and can also be expressed in circulating blood cells induced by stimulus (21, 28). During sepsis, invading pathogens encounter the innate immune system, and immune cells recognize pathogen-associated molecular patterns (PAMPs) or damage-associated molecular patterns (DAMPs) through pattern recognition receptors (PRRs) (29). In response to this, activated monocytes and endothelial cells, as well as their microparticles, express and release activated TF at pathogen exposure sites (30, 31). Moreover, the endothelial barrier injury during sepsis leads to TF exposure to coagulation factors in the blood (32). Cumulatively, TF initiates the extrinsic coagulation pathway and mediates the coagulation response in sepsis (24).

### 3.2 Neutrophil extracellular traps

Neutrophil extracellular traps (NETs) are DNA- and histone-based, web-like structures released by neutrophils to help capture pathogens (33). NETs promote inflammation and tissue damage during sepsis and support immunothrombosis in several ways (34). First, NETs can damage and activate endothelial cells (35). Due to their inherent ability to kill pathogens, the structures of NETs are extremely cytotoxic to host cells, damaging and killing endothelial cells, and leading to coagulation activation (36). NETs activate endothelial cells by inducing them to release adhering molecules and TF, and subsequently recruiting inflammatory cells and promoting inflammation and coagulation (35). Second, NETs and histones in NETs bind to vWF and promote the recruitment and activation of platelets (37–39). Third, NETs can promote the extrinsic pathway of coagulation by binding to TF, and the polyanionic surface of NETs activates the contact activation protein, factor XII, thereby activating the intrinsic pathway (40). Finally, neutrophil elastase and myeloperoxidase in NETs upregulate the procoagulant response through proteolytic cleavage and the oxidation of anticoagulants, such as thrombomodulin and TFPI (41, 42).

### 3.3 Platelets

Platelets are regarded as major actors in physiological hemostasis and are front-runners during sepsis (43). Currently, platelets are regarded as immune cells. During sepsis, platelets can be recruited and activated in a process closely resembling physiological hemostasis (44). Furthermore, when stimulated by PAMPs, DAMPs, and immune cells, platelets are activated by different platelet receptors such as glycoprotein Ib-V-IX, integrin αIIbβ3 (GPIIb/IIIa), and toll-like receptors (TLRs) (45). Once activated, platelets express adhesion receptors (for example, P-selection and CD 40 ligand) and release chemokines that recruit and interact with circulating leukocytes (43), amplifying “immunothrombosis”. In addition, platelet-derived polyphosphates activate factor XII, thereby initiating the intrinsic coagulation pathway (46).

### 3.4 Endothelial cells

The integrity of the endothelial barrier structure and function allows for antithrombotic function. The barrier function is maintained by the endothelial cytoskeleton, glycocalyx, intercellular adhesion molecules, and other proteins (47). During sepsis, endothelial activation and dysfunction are key events, that act as a bridge between the immune response and the coagulation cascade (47). Circulating PAMPs, DAMPs, and proinflammatory cytokines activate endothelial cells, which leads to the increased expression and release of adhesion molecules, mediating the adhesion and interactions of activated leukocytes and platelets (27, 48). Furthermore, injured or activated endothelial cells assume hypercoagulability with the release or expression of prothrombotic components, such as vWF and TF, and impairment of the membrane anticoagulant components, such as TFPI, thrombomodulin, and endothelial glycocalyx (27, 32).

### 3.5 Complements and cytokines

The complement system consists of numerous plasma and membrane-bound proteins, and functions as an intravascular surveillance system by killing pathogens (49). It is an essential component of the innate and adaptive immune systems (49). Complement activation, including C3a and C5a, also promotes coagulation and mediates the activation of endothelial cells and platelets (50, 51), and impairs the fibrinolytic system by increasing plasminogen activator inhibitor-1 (PAI-1) (52). Inflammation is triggered by the activation of innate immune cells, releasing proinflammatory cytokines such as tumor necrosis factor (TNF), interleukin‐1β (IL‐1β), IL-6, IL‐12, and IL‐18 (53). Cytokines are the most important mediators of hemostasis activation during sepsis, which are mainly mediated by increased expression of TF (24). In addition, cytokines can further activate leukocytes, platelets, and endothelial cells, thereby promoting hemostasis activation (54). Notably, these cytokines not only activate procoagulant pathways and platelets but also downregulate the anticoagulant pathways of TFPI, protein C system, and AT, along with impairing fibrinolysis pathways through a sustained increase in PAI-1 (23, 24, 55, 56).

Collectively, these immune mechanisms result in a procoagulant state in the hemostatic balance during sepsis. More importantly, the activation of hemostasis also feeds back to the immune response (3). For example, coagulation proteases bind to protease-activated receptors expressed in innate immune cells, which can induce additional proinflammatory responses by releasing cytokines (23, 57). In this section, we focus on immune-related factors that induce hemostatic changes in sepsis; a more comprehensive discussion of these changes is not included in this review.

## 4 Pyroptosis-induced hemostasis activation in sepsis

In the early stages of sepsis, the host defense reaction induces pyroptosis, which participates in the innate immune response, and eliminates intracellular pathogens. However, excessive pyroptosis results in a dysregulated host immune response, systemic inflammatory reactions, and even organ failure (7). In addition, recent studies have revealed that the activation of pyroptosis and inflammasome are widely involved in hemostasis activation and immunothrombosis in sepsis.

### 4.1 Mechanisms of pyroptosis

The phenomenon of pyroptosis was discovered in 1992 (58), which reported that the death of macrophages infected with *Shigella flexneri* was caspase-1 dependent. In 2001, the concept of “pyroptosis” was first coined by Cookson and Brennan (59), who described the proinflammatory and lytic mode of programmed cell death. Pyroptosis is stimulated by PAMPs, such as LPS, flagellin, and type 3 secretion system (T3SS) structural proteins, and DAMPs, such as ATP, uric acid crystals, and high-mobility group box 1 (HMGB1) (60). Pyroptosis mechanisms include the canonical (caspase-1 dependent) and non-canonical pathways (caspase-4/5/11 dependent) (60–62).

In the canonical pyroptosis pathway, an inflammasome complex is formed. Five intracellular PRRs have been confirmed to form inflammasomes: the nucleotide-binding oligomerization domain (NOD) and leucine-rich repeat-containing receptor (NLR) proteins, NLRP3 (NLR family pyrin domain containing 3), NLRP1, NLRC4 (NLR family caspase activation and recruitment domain [CARD] containing 4), as well as AIM2 (absent in melanoma 2) and pyrin (60, 63). Intracellular PRRs recognize pathogenic stimuli and bind to pro-caspase-1 through the adaptor protein apoptosis-associated speck-like protein containing a CARD (ASC), and assemble into inflammasome complexes that activate caspase-1 and then cleave pro-IL-1β and pro-IL-18 (60). In these inflammasome complexes, NLRP1 and NLRC4 contain CARD domains, resulting in the ability to recruit pro-caspase 1 with or without ASC (64, 65). Significantly, the NLRP3 inflammasome is the most extensively studied inflammasome, and the assembly of the NLRP3 inflammasome requires two signals (60). The first is the priming signal, which entails the transcription of nuclear factor-κB (NF-κB)-mediated upregulation of pro-IL-1 along with NLRP3 (66). Stimulants for priming include ligands for TLRs, NLRs, and cytokine receptors (66). The second signal activates the NLRP3 inflammasome and is provided by ATP, potassium (K^+^) efflux, calcium signaling, cytosolic release of lysosomal cathepsins and reactive oxygen species (ROS), and certain bacterial toxins (60, 67).

The non-canonical pyroptosis is initiated after intracellular LPS directly binds and activates caspase-4/5 (in humans) or caspase-11 (in mice) (61, 62). Furthermore, intracellular LPS stimulation activates NLRP3 inflammasomes through the cleavage of the pannexin-1 channel and the release of ATP which causes P2X7 receptor activation and subsequent K^+^ efflux (68–70). Hence, the NLRP3 inflammasome is a pivotal connection between the canonical and non-canonical pathways of pyroptosis.

Subsequently, the activated caspases 1/4/5/11 cleave gasdermin D (GSDMD) into the C-terminal (22kDa) and N-terminal (31kDa) (71). The N-terminal of GSDMD then migrates and inserts itself into the cell membrane, oligomerizes, and forms pores (71). IL-1β and IL-18 are cleaved into mature forms by caspase-1 and then released through the pores, which is additionally mediated by electrostatic filtering (71, 72). Pyroptotic cell death occurs through osmotic lysis, followed by the release of cell contents and a cascade of downstream responses (71, 73).

In addition to the canonical and non-canonical pathways of pyroptosis, new mechanisms triggering pyroptosis have been discovered. Caspase-3, a key marker of apoptosis activated by TNF-α or chemotherapy drugs, can mediate pyroptosis by cleaving gasdermin E (GSDME) and forming pores on the cell membrane (74). Moreover, Caspase-8 cleaves GSDMD and GSDME, which are also involved in pyroptosis (75, 76).

### 4.2 Pyroptosis mediates the hemostasis activation in sepsis

Pyroptosis has displayed a prominent role in hemostasis imbalance and “immunothrombosis” in sepsis: it participates in regulating the release and activity of TF in macrophages and endothelial cells; GSDMD mediates NETs formation; pyroptosis in endothelial cells and platelets affects hemostasis; the release of proinflammatory cytokines promotes hemostasis activation.

#### 4.2.1 Control of TF by pyroptosis

**Figure 3:**
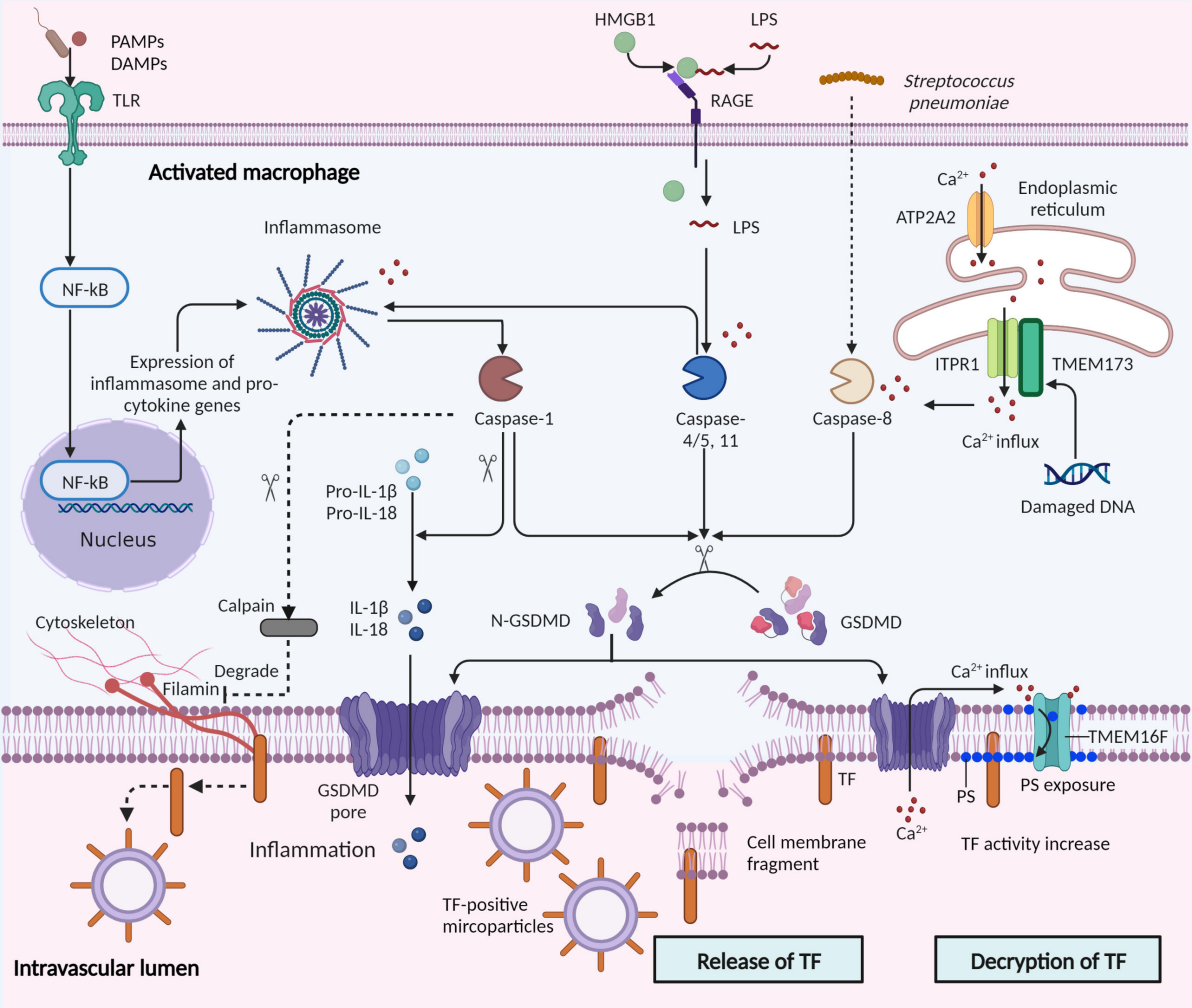
Control of tissue factor by pyroptosis in sepsis. Pyroptosis is stimulated by microbial infections and non-infectious stimuli, including host factors in sepsis. Canonical and non-canonical pyroptosis are both involved in tissue factor (TF) control. In canonical pyroptosis, pattern recognition receptors (PRRs) recognize pathogenic stimuli, and inflammasome complexes are assembled, which activate caspase-1 and then cleave pro-IL-1β and pro-IL-18. Intracellular LPS directly binds and activates caspase-11. Subsequently, the activated caspases-1/11 cleave the gasdermin D (GSDMD) into the N-terminal of GSDMD (N-GSDMD), forming pores in the cell membrane. IL-1β and IL-18 are then released through the pores and pyroptotic cell death occurs through osmotic lysis. TF is released in microparticles by the osmotic cell rupture in pyroptotic macrophages. In addition, caspase-1 may mediate TF release in another mechanism: activation of caspase-1 facilitates activation of calpain, degrading filamin in macrophages and thereby severing bonds between the cytoskeleton and TF. Furthermore, activation of transmembrane protein 173 (TMEM173) by infection-induced DNA damage mediates TF release. TMEM173 binds to inositol 1,4,5-trisphosphate receptor type 1 (ITPR1) in macrophages and monocytes, regulated the calcium influx, and activated GSDMD by caspase-1/11 or caspase-8.The GSDMD cleavage then triggers TF release through pyroptosis. Decryption of TF, a process increasing the procoagulant activity of TF, is triggered by GSDMD pores and subsequent phosphatidylserine (PS) exposure. GSDMD pores triggered calcium influx into the cytosol and promoted the exposure of PS to the outer cell membrane through TMEM16F, a calcium-dependent phospholipid scramblase. PAMPs, Pathogen-associated molecular patterns; DAMPs, damage-associated molecular patterns; TLR, toll-like receptors; NF-κB, nuclear factor-κB; RAGE, receptor for advanced glycation end products; ATP2A2, ATPase sarcoplasmic/ER Ca2+ transporting 2.

TF, the initiator of the extrinsic coagulation pathway, is a molecule prominently involved in the activation of pyroptosis-mediated coagulation in sepsis (10, 11, 77). Pyroptosis upregulates the release and activity of TF in sepsis, and the mechanisms are summarized in **Figure 3**.

***Release of TF:*** Wu et al. showed that macrophage-derived microparticles containing TF are released after inflammasome activation and pyroptosis (11). They found that the activation of caspase-1 by T3SS rod proteins from *Escherichia coli* (*E. coli*) or activation of caspase-11 by intracellular LPS lead to GSDMD cleavage and pore formation, and subsequently triggered pyroptotic death and osmotic cell rupture. The TF then released in macrophages-derived microparticles results in the systemic activation of coagulation and subsequent death in mice (11). The caspase-1/11 activated mice showed increased coagulation time and thrombin-antithrombin complex level, and decreased fibrinogen and platelet counts, consistent with DIC in sepsis (11). Additionally, Rothmeier et al. reported that activated caspase-1 induced TF release. They found that in ATP-triggered macrophages, inflammasomes induced the activation of intracellular caspase-1 and calpain cysteine protease cascade, degrading filamin in macrophages and thereby severing bonds between the cytoskeleton and tissue factor (78). Furthermore, a recent study showed that in a bacterial infection mouse model (cecal ligation and puncture, CLP), activation of transmembrane protein 173 (TMEM173, also known as stimulator of interferon response cGAMP interactor, [STING]) triggered TF release and mediated coagulation activation, which relied on calcium release from the endoplasmic reticulum (77). Mechanistically, TMEM173 binding to inositol 1,4,5-trisphosphate receptor type 1 (ITPR1, also known as IP3R, the primary calcium release channel of the endoplasmic reticulum) in macrophages and monocytes stimulated with *E. coli* and *Streptococcus pneumoniae*, regulated the calcium influx, activated GSDMD by caspase-1/11 or caspase-8, culminating in TF release *via* pyroptosis (77). Collectively, these findings highlight that pyroptosis-involved caspase-1/8/11 and the GSDMD system play central roles in TF release.

***Decryption of TF:*** Under physiological conditions, TF has low procoagulant activity, especially when expressed by blood cells and microparticles (79, 80). The decryption of TF is a post-translational process, increasing the procoagulant activity of TF by up to 100-fold (79, 81). This procoagulant phenotype characterizes TF-triggered “immunocoagulation”. One theory suggests that decryption is related to alterations of negatively charged phospholipids, such as phosphatidylserine, in the cell membrane (79). Recently, the decryption of TF has been shown to be related to inflammasome activation and pyroptosis. Yang et al. found that LPS activated caspase-11 and enhanced the decryption of TF by triggering the formation of GSDMD pores and subsequent phosphatidylserine (PS) exposure. Mechanistically, GSDMD pores triggered calcium influx into the cytosol and promoted the exposure of PS to the outer cell membrane through TMEM16F, a calcium-dependent phospholipid scramblase (10). Of note, the authors further utilized glycine and mannitol to prevent cell membrane rupture and cell lysis, and found that these did not affect TF’s procoagulant activity, suggesting that GSDMD-mediated TF decryption was independent of cell rupture in pyroptosis (10). In another study, high mobility group box 1 (HMGB1) from hepatocytes delivered extracellular LPS into the cytosol of macrophages and endothelial cells, where LPS induced caspase-11-dependent pyroptosis (82). Additionally, HMGB1 induced TF activation by promoting PS exposure through LPS-induced caspase-11 activation (83).

#### 4.2.2 GSGMD in NETs formation

As we mentioned above, NETs contribute to immunothrombosis in sepsis in multiple ways. Previous studies demonstrated that NETs release involved two ways: NETosis, a novel neutrophil cell death pathway (84), and DNA extrusion mechanism from live cells (85). Recently, new research indicated that GSDMD pore also promoted NETs formation and release. Chen et al. found that pyroptosis induction in neutrophils and the non-canonical pyroptosis pathway activated NETs formation (86). Caspase-11 and GSDMD mediated NETs release which was dependent on neutrophil plasma membrane rupture and did not require factors usually associated with NETs release. Caspase-11 and GSDMD also participated in early features of NETosis, which was mediated by the nuclear membrane permeabilization and histone degradation driven by caspase-11 and GSDMD (86). Interestingly, a different mechanism of GSDMD-mediated NETs was found by Sollberger et al (87). The researchers discovered that GSDMD was proteolytically activated by neutrophil proteases and, in turn, affected protease activation and nuclear expansion, which suggested that NETosis promoted by GSDMD was independent of inflammasome and caspase activation.

#### 4.2.3 Pyroptosis in endothelial cell activation and dysfunction

Pyroptosis can occur in many cell types during sepsis, including endothelial cells, the major targets of inflammatory mediators, and innate immune cells. LPS induces non-canonical pyroptotic death of endothelial cells through caspase-11, resulting in endothelial injury associated with vascular leakage, increased leukocyte accumulation, and cytokine release in the lungs (88). Moreover, pyroptotic endothelial cells release microparticles in a caspase-11-dependent manner (88). Circulating histones, as DAMPs in sepsis, have been reported to induce pyroptosis through the NLRP3 inflammasome in human umbilical vein endothelial cells, which further results in the expression of endothelial adhesion molecules and an inflammatory response (89). Circulatory exosomes from COVID-19 patients trigger the NLRP3 inflammasome in cultured endothelial cells (90). Furthermore, Wang et al. found that monocytic microparticles activate endothelial cells *via* NLRP3 inflammasome, which induces phosphorylation of ERK1/2, activation of the NF-κB pathway, and expression of the following cell adhesion molecules: intercellular adhesion molecule-1, vascular cell adhesion molecule-1, and E-selectin (91). These studies suggest that inflammasome activation and pyroptosis of endothelial cells induce endothelial activation and dysfunction, subsequently leading to inflammation and hemostasis activation.

#### 4.2.4 Inflammasome activation in platelets

Although anucleate, platelets have a functional spliceosome and essential spliceosome factors, that are associated with constitutively present mRNA transcripts, including IL-1β (92). Inflammasome activation in platelets shows “immunothrombosis” activities, which are mediated by IL-1β production (93). Hottz et al. demonstrated that dengue virus infection leads to the activation of NLRP3 inflammasomes in platelets, triggering platelet shedding of IL-1β-rich microparticles and increased expression of IL-1β, which contributes to increased endothelial permeability (94). The platelet NLRP3 inflammasome is upregulated during platelet activation and thrombus formation *in vitro (* 95); NLRP3 deficient platelets impaired hemostasis and thrombosis in mice (96). NLRP3 affected platelet αIIbβ3 outside-in signal transduction, an essential process for hemostasis and thrombosis, which might be mediated by IL-1β, as significantly reduced IL-1β release was found in NLRP3-deficient platelets (96). Furthermore, in thrombin activated-platelets, the caspase-3/GSDME pathway was upregulated, further regulating platelet integrin αIIbβ3 activation (97).

#### 4.2.5 Pyroptosis-released cytokines in hemostasis activation

During pyroptosis, IL-1β and IL-18, both important inflammatory cytokines in sepsis, are released (98). These cytokines, together with the downstream inflammatory mediators, such as IL-6 and TNF-α, further result in coagulation upregulation, leukocyte activation, endothelial activation and dysfunction, and platelets activation and aggregation (23, 54, 99–101). All these responses cumulatively lead to hemostasis activation and thrombus formation, accelerating the development of DIC in sepsis.

### 4.3 Potential therapeutic strategies targeting pyroptosis

Although excessive hemostasis activation in sepsis is associated with organ dysfunction and death, there have been long-standing debates over the efficacy of anticoagulant therapy in managing sepsis (102, 103), regarding the timing and the hemorrhagic complications. Theoretically, the most logical timing for anticoagulant treatment is the prothrombotic stage of the hemostasis change in sepsis. Inappropriate hemostasis intervention during “immunocoagulation” in host defense against pathogens, or during fibrinolytic DIC, will lead to the aggravation of sepsis, which should also be taken into consideration in the intervention of pyroptosis-induced hemostasis imbalance. Based on the mechanism of pyroptosis-induced hemostasis activation described earlier, potential inhibitors that may prevent extensive hemostasis activation in sepsis are listed below

#### 4.3.1 Inhibition of inflammasome activation

Previous studies have shown that inhibition of NLRP3 inflammasome activation results in protective effects against sepsis (104). In particular, MCC950, which inhibits NLRP3 by directly targeting the NLRP3 ATP-hydrolysis motif (105), attenuates multi-organ injuries in CLP rats (106), highlighting the potential of NLRP3 inhibitors in treating sepsis. Oridonin (107), tranilast (108), CY-09 (109), itaconate (110), etc. are further compounds that target NLRP3.

#### 4.3.2 Caspase-related inhibitors

Broad-spectrum caspase inhibitors Z-VAD-FMK and VX-166 can reduce IL-1β and IL-18 release and show significant therapeutic effects against sepsis in human patients and rodent models (111, 112). Ac-FLTD-CMK inhibits GSDMD cleavage by caspases-1/4/5/11, thereby suppressing canonical and non-canonical pyroptosis (113). Z-IETD-FMK blocks caspase-8-mediated GSDMD cleavage and prevents TF release in bone-marrow-derived macrophages (BMDMs) (77). oxPAPC, a caspase-4/11-targeting inhibitor, competes with LPS binding and consequently inhibits LPS-induced pyroptosis and septic shock (114). Ac-YVAD-CMK blocks caspase-1 activity, inhibiting IL-1β release and the generation of thrombo-inflammatory microparticles in BMDMs (78).

#### 4.3.3 Inhibition of GSDMD

Disulfiram, a drug used to treat alcohol addiction, as an inhibitor of pore formation by GSDMD, blocks pyroptosis and cytokine release in cells and prevents LPS-induced death in mice (115). LDC7559, a small molecule based on the pyrazolo-oxazepine scaffold, specifically binds GSDMD and blocks the activity of the GSDMD N-terminal (87).

In addition, blockers of other molecules or events regulating upstream and downstream inflammasome activation or pyroptosis also exhibit potential roles for the control of coagulation in sepsis (116–118). These include preventing pyroptosis-induced membrane rupture, inhibiting calcium influx, and preventing cytosolic delivery of LPS with heparin or by antagonizing HMGB1 (116–118). Although may be potential treatments for sepsis, unfortunately, they have not been translated into clinical sepsis, and more research is needed to convert promising experimental results into effective clinical drugs.

## 5 Conclusion

Sepsis is characterized by excessive hemostasis activation, multiple organ dysfunction, and high mortality, and remains a major challenge for basic and clinical research. Understanding the mechanisms of pyroptosis-induced hemostatic imbalance in sepsis increases our understanding of the characteristics and consequences of the host defense response to pathogens and brings new insights into sepsis-induced coagulopathy. The regulation of TF release and decryption in pyroptosis is a key discovery in “immunocoagulation,” as it links the key effectors of inflammation and coagulation. In addition, pyroptosis and inflammasome activation pathways contribute to NETs formation and activation of endothelial cells and platelet, which are related to hemostasis activation in sepsis. Elucidation of pyroptosis-induced hemostatic activation pathways and their implications in thrombosis might reveal new therapeutic approaches for septic DIC and lethal sepsis.

However, concerns and questions remain unaddressed. The pyroptosis pathways in hemostatic change are interconnected with other events in the development of sepsis, which means that intervention during hemostasis will affect the host response and could aggravate the disease. Hence, the focus should be on further in-depth comprehension of intermediate connections to achieve precise coagulation therapy. Moreover, proper therapies tailored to the prothrombotic stage of DIC should be established in the future.

## Author contributions

CZ and YLu reviewed the literature, wrote the draft of the manuscript, and prepared figures. YLi helped in the evaluation of the literature. XM designed and supervised the work. All authors contributed to the review and approved the submitted version. The research was supported by the National Nature Science Foundation of China (Grant No.82172130) and Program of Education Department of Liaoning Province (LJKMZ20221144).

## References

[1] Singer M Deutschman CS Seymour CW Shankar-Hari M Annane D Bauer M. The third international consensus definitions for sepsis and septic shock (Sepsis-3). JAMA (2016) 315(8):801–10. doi: 10.1001/jama.2016.0287 PMC4968574 26903338

[2] Rudd KE Johnson SC Agesa KM Shackelford KA Tsoi D Kievlan DR. Global, regional, and national sepsis incidence and mortality, 1990-2017: analysis for the global burden of disease study. Lancet (2020) 395(10219):200–11. doi: 10.1016/S0140-6736(19)32989-7 PMC6970225 31954465

[3] Engelmann B Massberg S. Thrombosis as an intravascular effector of innate immunity. Nat Rev Immunol (2013) 13(1):34–45. doi: 10.1038/nri3345 23222502

[4] Gando S Shiraishi A Yamakawa K Ogura H Saitoh D Fujishima S. Role of disseminated intravascular coagulation in severe sepsis. Thromb Res (2019) 178:182–8. doi: 10.1016/j.thromres.2019.04.025 31054468

[5] Miao EA Leaf IA Treuting PM Mao DP Dors M Sarkar A. Caspase-1-induced pyroptosis is an innate immune effector mechanism against intracellular bacteria. Nat Immunol (2010) 11(12):1136–42. doi: 10.1038/ni.1960 PMC3058225 21057511

[6] Aachoui Y Leaf IA Hagar JA Fontana MF Campos CG Zak DE. Caspase-11 protects against bacteria that escape the vacuole. Science (2013) 339(6122):975–8. doi: 10.1126/science.1230751 PMC3697099 23348507

[7] Aglietti RA Dueber EC. Recent insights into the molecular mechanisms underlying pyroptosis and gasdermin family functions. Trends Immunol (2017) 38(4):261–71. doi: 10.1016/j.it.2017.01.003 28196749

[8] Aziz M Jacob A Wang P. Revisiting caspases in sepsis. Cell Death disease (2014) 5:e1526. doi: 10.1038/cddis.2014.488 25412304 PMC4260746

[9] Zheng X Chen W Gong F Chen Y Chen E. The role and mechanism of pyroptosis and potential therapeutic targets in sepsis: A review. Front Immunol (2021) 12:711939. doi: 10.3389/fimmu.2021.711939 34305952 PMC8293747

[10] Yang X Cheng X Tang Y Qiu X Wang Y Kang H. Bacterial endotoxin activates the coagulation cascade through gasdermin d-dependent phosphatidylserine exposure. Immunity (2019) 51(6):983–96.e6. doi: 10.1016/j.immuni.2019.11.005 31836429

[11] Wu C Lu W Zhang Y Zhang G Shi X Hisada Y. Inflammasome activation triggers blood clotting and host death through pyroptosis. Immunity (2019) 50(6):1401–11.e4. doi: 10.1016/j.immuni.2019.04.003 31076358 PMC6791531

[12] Furie B Furie BC. Mechanisms of thrombus formation. New Engl J Med (2008) 359(9):938–49. doi: 10.1056/NEJMra0801082 18753650

[13] Wilcox JN Smith KM Schwartz SM Gordon D. Localization of tissue factor in the normal vessel wall and in the atherosclerotic plaque. Proc Natl Acad Sci U S A (1989) 86(8):2839–43. doi: 10.1073/pnas.86.8.2839 PMC287014 2704749

[14] Moog S Mangin P Lenain N Strassel C Ravanat C Schuhler S. Platelet glycoprotein V binds to collagen and participates in platelet adhesion and aggregation. Blood (2001) 98(4):1038–46. doi: 10.1182/blood.V98.4.1038 11493449

[15] Bergmeier W Chauhan AK Wagner DD. Glycoprotein ibalpha and von willebrand factor in primary platelet adhesion and thrombus formation: lessons from mutant mice. Thromb haemostasis (2008) 99(2):264–70. doi: 10.1160/TH07-10-0638 18278173

[16] Dubois C Panicot-Dubois L Gainor JF Furie BC Furie B. Thrombin-initiated platelet activation *in vivo* is vWF independent during thrombus formation in a laser injury model. J Clin Invest (2007) 117(4):953–60. doi: 10.1172/JCI30537 PMC1821068 17380206

[17] Vu TK Hung DT Wheaton VI Coughlin SR. Molecular cloning of a functional thrombin receptor reveals a novel proteolytic mechanism of receptor activation. Cell (1991) 64(6):1057–68. doi: 10.1016/0092-8674(91)90261-V 1672265

[18] Davie EW Ratnoff OD. Waterfall sequence for intrinsic blood clotting. Science (1964) 145(3638):1310–2. doi: 10.1126/science.145.3638.1310 14173416

[19] Macfarlane RG. An enzyme cascade in the blood clotting mechanism, and its function as a biochemical amplifier. Nature (1964) 202:498–9. doi: 10.1038/202498a0 14167839

[20] Josso F Prou-Wartelle O. Interaction of tissue factor and factor VII at the earliest phase of coagulation. Thromb Diath Haemorrh Suppl (1965) 17:35–44. 5874847

[21] Witkowski M Landmesser U Rauch U. Tissue factor as a link between inflammation and coagulation. Trends Cardiovasc Med (2016) 26(4):297–303. doi: 10.1016/j.tcm.2015.12.001 26877187

[22] Luchtman-Jones L Broze GJ Jr. The current status of coagulation. Ann Med (1995) 27(1):47–52. doi: 10.3109/07853899509031935 7741998

[23] Levi M van der Poll T. Inflammation and coagulation. Crit Care Med (2010) 38(2 Suppl):S26–34. doi: 10.1097/CCM.0b013e3181c98d21 20083910

[24] Amaral A Opal SM Vincent JL. Coagulation in sepsis. Intensive Care Med (2004) 30(6):1032–40. doi: 10.1007/s00134-004-2291-8 15148567

[25] van der Poll T van de Veerdonk FL Scicluna BP Netea MG. The immunopathology of sepsis and potential therapeutic targets. Nat Rev Immunol (2017) 17(7):407–20. doi: 10.1038/nri.2017.36 28436424

[26] Levi M Opal SM. Coagulation abnormalities in critically ill patients. Crit Care (2006) 10(4):222. doi: 10.1186/cc4975 16879728 PMC1750988

[27] Iba T Levy JH. Inflammation and thrombosis: roles of neutrophils, platelets and endothelial cells and their interactions in thrombus formation during sepsis. J Thromb haemostasis JTH (2018) 16(2):231–41. doi: 10.1111/jth.13911 29193703

[28] Semeraro N Biondi A Lorenzet R Locati D Mantovani A Donati MB. Direct induction of tissue factor synthesis by endotoxin in human macrophages from diverse anatomical sites. Immunology (1983) 50(4):529–35. PMC1454387 6654386

[29] Tang D Kang R Coyne CB Zeh HJ Lotze MT. PAMPs and DAMPs: signal 0s that spur autophagy and immunity. Immunol Rev (2012) 249(1):158–75. doi: 10.1111/j.1600-065X.2012.01146.x PMC3662247 22889221

[30] Osterud B. Tissue factor expression by monocytes: regulation and pathophysiological roles. Blood Coagul Fibrinolysis (1998) 9 Suppl 1:S9–14. 9819023

[31] Osterud B. Tissue factor expression in blood cells. Thromb Res (2010) 125 Suppl 1:S31–4. doi: 10.1016/j.thromres.2010.01.032 20149415

[32] Ince C Mayeux PR Nguyen T Gomez H Kellum JA Ospina-Tascon GA. The endothelium in sepsis. Shock (2016) 45(3):259–70. doi: 10.1097/SHK.0000000000000473 PMC5281063 26871664

[33] Brinkmann V Reichard U Goosmann C Fauler B Uhlemann Y Weiss DS. Neutrophil extracellular traps kill bacteria. Science (2004) 303(5663):1532–5. doi: 10.1126/science.1092385 15001782

[34] Martinod K Wagner DD. Thrombosis: tangled up in NETs. Blood (2014) 123(18):2768–76. doi: 10.1182/blood-2013-10-463646 PMC4007606 24366358

[35] Aldabbous L Abdul-Salam V McKinnon T Duluc L Pepke-Zaba J Southwood M. Neutrophil extracellular traps promote angiogenesis: Evidence from vascular pathology in pulmonary hypertension. Arteriosclerosis thrombosis Vasc Biol (2016) 36(10):2078–87. doi: 10.1161/ATVBAHA.116.307634 27470511

[36] Saffarzadeh M Juenemann C Queisser MA Lochnit G Barreto G Galuska SP. Neutrophil extracellular traps directly induce epithelial and endothelial cell death: a predominant role of histones. PLoS One (2012) 7(2):e32366. doi: 10.1371/journal.pone.0032366 22389696 PMC3289648

[37] Fuchs TA Brill A Duerschmied D Schatzberg D Monestier M Myers DD Jr.. Extracellular DNA traps promote thrombosis. Proc Natl Acad Sci U S A (2010) 107(36):15880–5. doi: 10.1073/pnas.1005743107 PMC2936604 20798043

[38] Brill A Fuchs TA Savchenko AS Thomas GM Martinod K De Meyer SF. Neutrophil extracellular traps promote deep vein thrombosis in mice. J Thromb haemostasis JTH (2012) 10(1):136–44. doi: 10.1111/j.1538-7836.2011.04544.x PMC3319651 22044575

[39] Ward CM Tetaz TJ Andrews RK Berndt MC. Binding of the von willebrand factor A1 domain to histone. Thromb Res (1997) 86(6):469–77. doi: 10.1016/S0049-3848(97)00096-0 9219327

[40] von Bruhl ML Stark K Steinhart A Chandraratne S Konrad I Lorenz M. Monocytes, neutrophils, and platelets cooperate to initiate and propagate venous thrombosis in mice in vivo. J Exp Med (2012) 209(4):819–35. doi: 10.1084/jem.20112322 PMC3328366 22451716

[41] Massberg S Grahl L von Bruehl ML Manukyan D Pfeiler S Goosmann C. Reciprocal coupling of coagulation and innate immunity *via* neutrophil serine proteases. Nat Med (2010) 16(8):887–96. doi: 10.1038/nm.2184 20676107

[42] Glaser CB Morser J Clarke JH Blasko E McLean K Kuhn I. Oxidation of a specific methionine in thrombomodulin by activated neutrophil products blocks cofactor activity. A potential Rapid Mech modulation coagulation J Clin Invest (1992) 90(6):2565–73. doi: 10.1172/JCI116151 PMC443416 1334978

[43] Semple JW Italiano JE Jr. Freedman J. Platelets and the immune continuum. Nat Rev Immunol (2011) 11(4):264–74. doi: 10.1038/nri2956 21436837

[44] Davis RP Miller-Dorey S Jenne CN. Platelets and coagulation in infection. Clin Transl Immunol (2016) 5(7):e89. doi: 10.1038/cti.2016.39 PMC4973322 27525062

[45] Nicolai L Gaertner F Massberg S. Platelets in host defense: Experimental and clinical insights. Trends Immunol (2019) 40(10):922–38. doi: 10.1016/j.it.2019.08.004 31601520

[46] Muller F Mutch NJ Schenk WA Smith SA Esterl L Spronk HM. Platelet polyphosphates are proinflammatory and procoagulant mediators in vivo. Cell (2009) 139(6):1143–56. doi: 10.1016/j.cell.2009.11.001 PMC2796262 20005807

[47] Opal SM van der Poll T. Endothelial barrier dysfunction in septic shock. J Internal Med (2015) 277(3):277–93. doi: 10.1111/joim.12331 25418337

[48] Budnik I Brill A. Immune factors in deep vein thrombosis initiation. Trends Immunol (2018) 39(8):610–23. doi: 10.1016/j.it.2018.04.010 PMC6065414 29776849

[49] Barnum SR. Complement: A primer for the coming therapeutic revolution. Pharmacol Ther (2017) 172:63–72. doi: 10.1016/j.pharmthera.2016.11.014 27914981

[50] Eriksson O Mohlin C Nilsson B Ekdahl KN. The human platelet as an innate immune cell: Interactions between activated platelets and the complement system. Front Immunol (2019) 10:1590. doi: 10.3389/fimmu.2019.01590 31354729 PMC6635567

[51] Oikonomopoulou K Ricklin D Ward PA Lambris JD. Interactions between coagulation and complement–their role in inflammation. Semin Immunopathol (2012) 34(1):151–65. doi: 10.1007/s00281-011-0280-x PMC3372068 21811895

[52] Wojta J Kaun C Zorn G Ghannadan M Hauswirth AW Sperr WR. C5a stimulates production of plasminogen activator inhibitor-1 in human mast cells and basophils. Blood (2002) 100(2):517–23. doi: 10.1182/blood.V100.2.517 12091343

[53] Chousterman BG Swirski FK Weber GF. Cytokine storm and sepsis disease pathogenesis. Semin Immunopathol (2017) 39(5):517–28. doi: 10.1007/s00281-017-0639-8 28555385

[54] Schulte W Bernhagen J Bucala R. Cytokines in sepsis: potent immunoregulators and potential therapeutic targets–an updated view. Mediators inflammation (2013) 2013:165974. doi: 10.1155/2013/165974 PMC3703895 23853427

[55] Nawroth PP Stern DM. Modulation of endothelial cell hemostatic properties by tumor necrosis factor. J Exp Med (1986) 163(3):740–5. doi: 10.1084/jem.163.3.740 PMC2188058 3753996

[56] Kang S Tanaka T Inoue H Ono C Hashimoto S Kioi Y. IL-6 trans-signaling induces plasminogen activator inhibitor-1 from vascular endothelial cells in cytokine release syndrome. Proc Natl Acad Sci U S A (2020) 117(36):22351–6. doi: 10.1073/pnas.2010229117 PMC7486751 32826331

[57] Niessen F Schaffner F Furlan-Freguia C Pawlinski R Bhattacharjee G Chun J. Dendritic cell PAR1-S1P3 signalling couples coagulation and inflammation. Nature (2008) 452(7187):654–8. doi: 10.1038/nature06663 18305483

[58] Zychlinsky A Prevost MC Sansonetti PJ. Shigella flexneri induces apoptosis in infected macrophages. Nature (1992) 358(6382):167–9. doi: 10.1038/358167a0 1614548

[59] Cookson BT Brennan MA. Pro-inflammatory programmed cell death. Trends Microbiol (2001) 9(3):113–4. doi: 10.1016/S0966-842X(00)01936-3 11303500

[60] Lamkanfi M Dixit VM. Mechanisms and functions of inflammasomes. Cell (2014) 157(5):1013–22. doi: 10.1016/j.cell.2014.04.007 24855941

[61] Kayagaki N Stowe IB Lee BL O'Rourke K Anderson K Warming S. Caspase-11 cleaves gasdermin d for non-canonical inflammasome signalling. Nature (2015) 526(7575):666–71. doi: 10.1038/nature15541 26375259

[62] Shi J Zhao Y Wang Y Gao W Ding J Li P. Inflammatory caspases are innate immune receptors for intracellular LPS. Nature (2014) 514(7521):187–92. doi: 10.1038/nature13683 25119034

[63] Broz P Dixit VM. Inflammasomes: mechanism of assembly, regulation and signalling. Nat Rev Immunol (2016) 16(7):407–20. doi: 10.1038/nri.2016.58 27291964

[64] Proell M Riedl SJ Fritz JH Rojas AM Schwarzenbacher R. The nod-like receptor (NLR) family: a tale of similarities and differences. PLoS One (2008) 3(4):e2119. doi: 10.1371/journal.pone.0002119 18446235 PMC2323615

[65] Broz P von Moltke J Jones JW Vance RE Monack DM. Differential requirement for caspase-1 autoproteolysis in pathogen-induced cell death and cytokine processing. Cell Host Microbe (2010) 8(6):471–83. doi: 10.1016/j.chom.2010.11.007 PMC3016200 21147462

[66] Bauernfeind FG Horvath G Stutz A Alnemri ES MacDonald K Speert D. Cutting edge: NF-kappaB activating pattern recognition and cytokine receptors license NLRP3 inflammasome activation by regulating NLRP3 expression. J Immunol (2009) 183(2):787–91. doi: 10.4049/jimmunol.0901363 PMC2824855 19570822

[67] Franchi L Munoz-Planillo R Nunez G. Sensing and reacting to microbes through the inflammasomes. Nat Immunol (2012) 13(4):325–32. doi: 10.1038/ni.2231 PMC3449002 22430785

[68] Yang D He Y Munoz-Planillo R Liu Q Nunez G. Caspase-11 requires the pannexin-1 channel and the purinergic P2X7 pore to mediate pyroptosis and endotoxic shock. Immunity (2015) 43(5):923–32. doi: 10.1016/j.immuni.2015.10.009 PMC4795157 26572062

[69] Karmakar M Katsnelson M Malak HA Greene NG Howell SJ Hise AG. Neutrophil IL-1beta processing induced by pneumolysin is mediated by the NLRP3/ASC inflammasome and caspase-1 activation and is dependent on k+ efflux. J Immunol (2015) 194(4):1763–75. doi: 10.4049/jimmunol.1401624 PMC4369676 25609842

[70] Karmakar M Katsnelson MA Dubyak GR Pearlman E. Neutrophil P2X7 receptors mediate NLRP3 inflammasome-dependent IL-1beta secretion in response to ATP. Nat Commun (2016) 7:10555. doi: 10.1038/ncomms10555 26877061 PMC4756306

[71] Shi J Zhao Y Wang K Shi X Wang Y Huang H. Cleavage of GSDMD by inflammatory caspases determines pyroptotic cell death. Nature (2015) 526(7575):660–5. doi: 10.1038/nature15514 26375003

[72] Xia S Zhang Z Magupalli VG Pablo JL Dong Y Vora SM. Gasdermin d pore structure reveals preferential release of mature interleukin-1. Nature (2021) 593(7860):607–11. doi: 10.1038/s41586-021-03478-3 PMC8588876 33883744

[73] Bergsbaken T Fink SL Cookson BT. Pyroptosis: host cell death and inflammation. Nat Rev Microbiol (2009) 7(2):99–109. doi: 10.1038/nrmicro2070 19148178 PMC2910423

[74] Wang Y Gao W Shi X Ding J Liu W He H. Chemotherapy drugs induce pyroptosis through caspase-3 cleavage of a gasdermin. Nature (2017) 547(7661):99–103. doi: 10.1038/nature22393 28459430

[75] Orning P Weng D Starheim K Ratner D Best Z Lee B. Pathogen blockade of TAK1 triggers caspase-8-dependent cleavage of gasdermin d and cell death. Science (2018) 362(6418):1064–9. doi: 10.1126/science.aau2818 PMC6522129 30361383

[76] Sarhan J Liu BC Muendlein HI Li P Nilson R Tang AY. Caspase-8 induces cleavage of gasdermin d to elicit pyroptosis during yersinia infection. Proc Natl Acad Sci U S A (2018) 115(46):E10888–E97. doi: 10.1073/pnas.1809548115 PMC6243247 30381458

[77] Zhang H Zeng L Xie M Liu J Zhou B Wu R. TMEM173 drives lethal coagulation in sepsis. Cell Host Microbe (2020) 27(4):556–70.e6. doi: 10.1016/j.chom.2020.02.004 32142632 PMC7316085

[78] Rothmeier AS Marchese P Petrich BG Furlan-Freguia C Ginsberg MH Ruggeri ZM. Caspase-1-mediated pathway promotes generation of thromboinflammatory microparticles. J Clin Invest (2015) 125(4):1471–84. doi: 10.1172/JCI79329 PMC4396490 25705884

[79] Butenas S Krudysz-Amblo J. Decryption of tissue factor. Thromb Res (2012) 129 Suppl 2:S18–20. doi: 10.1016/j.thromres.2012.02.022 PMC3336021 22401800

[80] Morel O Toti F Hugel B Bakouboula B Camoin-Jau L Dignat-George F. Procoagulant microparticles: disrupting the vascular homeostasis equation? Arteriosclerosis thrombosis Vasc Biol (2006) 26(12):2594–604. doi: 10.1161/01.ATV.0000246775.14471.26 16990554

[81] Bach R Rifkin DB. Expression of tissue factor procoagulant activity: regulation by cytosolic calcium. Proc Natl Acad Sci U S A (1990) 87(18):6995–9. doi: 10.1073/pnas.87.18.6995 PMC54669 2119499

[82] Deng M Tang Y Li W Wang X Zhang R Zhang X. The endotoxin delivery protein HMGB1 mediates caspase-11-Dependent lethality in sepsis. Immunity (2018) 49(4):740–53.e7. doi: 10.1016/j.immuni.2018.08.016 30314759 PMC6300139

[83] Yang X Cheng X Tang Y Qiu X Wang Z Fu G. The role of type 1 interferons in coagulation induced by gram-negative bacteria. Blood (2020) 135(14):1087–100. doi: 10.1182/blood.2019002282 PMC7118812 32016282

[84] Fuchs TA Abed U Goosmann C Hurwitz R Schulze I Wahn V. Novel cell death program leads to neutrophil extracellular traps. J Cell Biol (2007) 176(2):231–41. doi: 10.1083/jcb.200606027 PMC2063942 17210947

[85] Pilsczek FH Salina D Poon KK Fahey C Yipp BG Sibley CD. A novel mechanism of rapid nuclear neutrophil extracellular trap formation in response to staphylococcus aureus. J Immunol (2010) 185(12):7413–25. doi: 10.4049/jimmunol.1000675 21098229

[86] Chen KW Monteleone M Boucher D Sollberger G Ramnath D Condon ND. Noncanonical inflammasome signaling elicits gasdermin d-dependent neutrophil extracellular traps. Sci Immunol (2018) 3(26). doi: 10.1126/sciimmunol.aar6676 30143554

[87] Sollberger G Choidas A Burn GL Habenberger P Di Lucrezia R Kordes S. Gasdermin d plays a vital role in the generation of neutrophil extracellular traps. Sci Immunol (2018) 3(26). doi: 10.1126/sciimmunol.aar6689 30143555

[88] Cheng KT Xiong S Ye Z Hong Z Di A Tsang KM. Caspase-11-mediated endothelial pyroptosis underlies endotoxemia-induced lung injury. J Clin Invest (2017) 127(11):4124–35. doi: 10.1172/JCI94495 PMC5663346 28990935

[89] Beltran-Garcia J Osca-Verdegal R Perez-Cremades D Novella S Hermenegildo C Pallardo FV. Extracellular histones activate endothelial NLRP3 inflammasome and are associated with a severe sepsis phenotype. J Inflammation Res (2022) 15:4217–38. doi: 10.2147/JIR.S363693 PMC9338392 35915852

[90] Sur S Steele R Isbell TS Ray R Ray RB. Circulatory exosomes from COVID-19 patients trigger NLRP3 inflammasome in endothelial cells. mBio (2022) 13(3):e0095122. doi: 10.1128/mbio.00951-22 35587188 PMC9239151

[91] Wang JG Williams JC Davis BK Jacobson K Doerschuk CM Ting JP. Monocytic microparticles activate endothelial cells in an IL-1beta-dependent manner. Blood (2011) 118(8):2366–74. doi: 10.1182/blood-2011-01-330878 PMC3162361 21700772

[92] Denis MM Tolley ND Bunting M Schwertz H Jiang H Lindemann S. Escaping the nuclear confines: signal-dependent pre-mRNA splicing in anucleate platelets. Cell (2005) 122(3):379–91. doi: 10.1016/j.cell.2005.06.015 PMC4401993 16096058

[93] Hottz ED Monteiro AP Bozza FA Bozza PT. Inflammasome in platelets: allying coagulation and inflammation in infectious and sterile diseases? Mediators Inflammation (2015) 2015:435783. doi: 10.1155/2015/435783 PMC4357129 25814789

[94] Hottz ED Lopes JF Freitas C Valls-de-Souza R Oliveira MF Bozza MT. Platelets mediate increased endothelium permeability in dengue through NLRP3-inflammasome activation. Blood (2013) 122(20):3405–14. doi: 10.1182/blood-2013-05-504449 PMC3829114 24009231

[95] Murthy P Durco F Miller-Ocuin JL Takedai T Shankar S Liang X. The NLRP3 inflammasome and bruton's tyrosine kinase in platelets co-regulate platelet activation, aggregation, and *in vitro* thrombus formation. Biochem Biophys Res Commun (2017) 483(1):230–6. doi: 10.1016/j.bbrc.2016.12.161 28034752

[96] Qiao J Wu X Luo Q Wei G Xu M Wu Y. NLRP3 regulates platelet integrin alphaIIbbeta3 outside-in signaling, hemostasis and arterial thrombosis. Haematologica (2018) 103(9):1568–76. doi: 10.3324/haematol.2018.191700 PMC6119128 29794149

[97] Li Y Xin G Li S Dong Y Zhu Y Yu X. PD-L1 regulates platelet activation and thrombosis *via* caspase-3/GSDME pathway. Front Pharmacol (2022) 13:921414. doi: 10.3389/fphar.2022.921414 35784685 PMC9240427

[98] Dinarello CA Novick D Kim S Kaplanski G. Interleukin-18 and IL-18 binding protein. Front Immunol (2013) 4:289. doi: 10.3389/fimmu.2013.00289 24115947 PMC3792554

[99] Chanchal S Mishra A Singh MK Ashraf MZ. Understanding inflammatory responses in the manifestation of prothrombotic phenotypes. Front Cell Dev Biol (2020) 8:73. doi: 10.3389/fcell.2020.00073 32117993 PMC7033430

[100] Levi M van der Poll T ten Cate H van Deventer SJ. The cytokine-mediated imbalance between coagulant and anticoagulant mechanisms in sepsis and endotoxaemia. Eur J Clin Invest (1997) 27(1):3–9. doi: 10.1046/j.1365-2362.1997.570614.x 9041370

[101] Szotowski B Antoniak S Poller W Schultheiss HP Rauch U. Procoagulant soluble tissue factor is released from endothelial cells in response to inflammatory cytokines. Circ Res (2005) 96(12):1233–9. doi: 10.1161/01.RES.0000171805.24799.fa 15920023

[102] Ranieri VM Thompson BT Barie PS Dhainaut JF Douglas IS Finfer S. Drotrecogin alfa (activated) in adults with septic shock. New Engl J Med (2012) 366(22):2055–64. doi: 10.1056/NEJMoa1202290 22616830

[103] Jaimes F de la Rosa G Morales C Fortich F Arango C Aguirre D. Unfractioned heparin for treatment of sepsis: A randomized clinical trial (The HETRASE study). Crit Care Med (2009) 37(4):1185–96. doi: 10.1097/CCM.0b013e31819c06bc 19242322

[104] Danielski LG Giustina AD Bonfante S Barichello T Petronilho F. The NLRP3 inflammasome and its role in sepsis development. Inflammation (2020) 43(1):24–31. doi: 10.1007/s10753-019-01124-9 31741197

[105] Coll RC Hill JR Day CJ Zamoshnikova A Boucher D Massey NL. MCC950 directly targets the NLRP3 ATP-hydrolysis motif for inflammasome inhibition. Nat Chem Biol (2019) 15(6):556–9. doi: 10.1038/s41589-019-0277-7 31086327

[106] Cornelius DC Travis OK Tramel RW Borges-Rodriguez M Baik CH Greer M. NLRP3 inflammasome inhibition attenuates sepsis-induced platelet activation and prevents multi-organ injury in cecal-ligation puncture. PloS One (2020) 15(6):e0234039. doi: 10.1371/journal.pone.0234039 32555710 PMC7299389

[107] He H Jiang H Chen Y Ye J Wang A Wang C. Oridonin is a covalent NLRP3 inhibitor with strong anti-inflammasome activity. Nat Commun (2018) 9(1):2550. doi: 10.1038/s41467-018-04947-6 29959312 PMC6026158

[108] Huang Y Jiang H Chen Y Wang X Yang Y Tao J. Tranilast directly targets NLRP3 to treat inflammasome-driven diseases. EMBO Mol Med (2018) 10(4). doi: 10.15252/emmm.201708689 PMC5887903 29531021

[109] Jiang H He H Chen Y Huang W Cheng J Ye J. Identification of a selective and direct NLRP3 inhibitor to treat inflammatory disorders. J Exp Med (2017) 214(11):3219–38. doi: 10.1084/jem.20171419 PMC5679172 29021150

[110] Hooftman A Angiari S Hester S Corcoran SE Runtsch MC Ling C. The immunomodulatory metabolite itaconate modifies NLRP3 and inhibits inflammasome activation. Cell Metab (2020) 32(3):468–78.e7. doi: 10.1016/j.cmet.2020.07.016 32791101 PMC7422798

[111] Oberholzer A Harter L Feilner A Steckholzer U Trentz O Ertel W. Differential effect of caspase inhibition on proinflammatory cytokine release in septic patients. Shock (2000) 14(3):253–7. doi: 10.1097/00024382-200014030-00002 11028539

[112] Weber P Wang P Maddens S Wang P Wu R Miksa M. VX-166: a novel potent small molecule caspase inhibitor as a potential therapy for sepsis. Crit Care (2009) 13(5):R146. doi: 10.1186/cc8041 19740426 PMC2784364

[113] Yang J Liu Z Wang C Yang R Rathkey JK Pinkard OW. Mechanism of gasdermin d recognition by inflammatory caspases and their inhibition by a gasdermin d-derived peptide inhibitor. Proc Natl Acad Sci U S A (2018) 115(26):6792–7. doi: 10.1073/pnas.1800562115 PMC6042100 29891674

[114] Chu LH Indramohan M Ratsimandresy RA Gangopadhyay A Morris EP Monack DM. The oxidized phospholipid oxPAPC protects from septic shock by targeting the non-canonical inflammasome in macrophages. Nat Commun (2018) 9(1):996. doi: 10.1038/s41467-018-03409-3 29520027 PMC5843631

[115] Hu JJ Liu X Xia S Zhang Z Zhang Y Zhao J. FDA-Approved disulfiram inhibits pyroptosis by blocking gasdermin d pore formation. Nat Immunol (2020) 21(7):736–45. doi: 10.1038/s41590-020-0669-6 PMC7316630 32367036

[116] Tang Y Wang X Li Z He Z Yang X Cheng X. Heparin prevents caspase-11-dependent septic lethality independent of anticoagulant properties. Immunity (2021) 54(3):454–67.e6. doi: 10.1016/j.immuni.2021.01.007 33561388

[117] Tang D Wang H Billiar TR Kroemer G Kang R. Emerging mechanisms of immunocoagulation in sepsis and septic shock. Trends Immunol (2021) 42(6):508–22. doi: 10.1016/j.it.2021.04.001 PMC8436187 33906793

[118] Wu R Wang N Comish PB Tang D Kang R. Inflammasome-dependent coagulation activation in sepsis. Front Immunol (2021) 12:641750. doi: 10.3389/fimmu.2021.641750 33796108 PMC8007875

